# Investigation of the Adsorption Behavior of Jet-Cooked Cationic Starches on Pulp Fibers

**DOI:** 10.3390/polym12102249

**Published:** 2020-09-29

**Authors:** Esther Ferstl, Martin Gabriel, Florian Gomernik, Stefanie Monika Müller, Julian Selinger, Ferula Thaler, Wolfgang Bauer, Frank Uhlig, Stefan Spirk, Angela Chemelli

**Affiliations:** 1 Institute of Inorganic Chemistry, Graz University of Technology, Stremayrgasse 9, 8010 Graz, Austria; esther.ferstl@student.tugraz.at (E.F.); ferula.thaler@gmail.com (F.T.); frank.uhlig@tugraz.at (F.U.); 2 Institute of Bioproducts and Paper Technology, Graz University of Technology, Inffeldgasse 23, 8010 Graz, Austria; martin.gabriel@student.tugraz.at (M.G.); florian.gomernik@vkbau.at (F.G.); stefanie.mueller@sbg.at (S.M.M.); julian.selinger@tugraz.at (J.S.); wolfgang.bauer@tugraz.at (W.B.); stefan.spirk@tugraz.at (S.S.); 3 Department of Bioproducts and Biosystems, Aalto University, P. O. Box 16300, 00076 Aalto, Finland

**Keywords:** steam-jet-cooking, cationic starch, adsorption on pulp fibers

## Abstract

The optimization of the thermal treatment of cationic starch in the paper industry offers the opportunity to reduce the energy consumption of this process. Four different industrially relevant cationic starches, varying in source, cationization method and degree of substitution were treated by a steam-jet cooking procedure, comparable to industrially employed starch cooking processes. The influence of the starch properties and cooking parameters on the adsorption behavior of the starches on cellulosic pulp was investigated. The adsorbed amount was affected by the cooking temperature and the type of starch. For some starch grades, a cooking temperature of 115 °C can be employed to achieve sufficient starch retention on the pulp fibers. The energy consumption could further be reduced by cooking at higher starch concentrations without loss of adsorption efficiency.

## 1. Introduction

Besides cellulosic fibers and mineral fillers, starch is one of the main components in paper-based products. In the paper industry, starch derived from corn, potato, wheat as well as tapioca is used, depending on the region. Starch consists of amylose, which has a mainly linear structure with few long chain branches, and of amylopectin, which has a high number of short-chain branches and higher molecular weights [1,2]. The ratio of amylose to amylopectin in starch depends on the botanical source. However, special varieties have been cultivated that have a very high proportion (up to 100%) of amylopectin and usually carry the term “waxy” together with its source, i.e., waxy potato starch.

Starch used in the wet-end of the paper machine as a dry strength and retention agent commonly contains cationic groups which have been introduced through chemical modification of the native starch [3,4]. The cationic functionalities improve the interaction with cellulosic fibers, enhancing inter-fiber bonding and resulting in enhanced dry strength of the paper [5,6,7,8,9]. Various synthetic methods have been employed to introduce cationic groups onto the starch [10,11]. Wet-cationization processes can be performed under either heterogeneous or homogenous conditions, depending on the solution state of the starch. Additionally, functionalization with cationic groups is also performed in (semi-)dry processes [12]. The advantages of these include reduced water usage, thereby reducing energy consumption for reaction conditioning/drying and avoiding the loss of starch. Cationic starches derived from wet or dry processes differ in their properties such as granular structure, dissolution properties, and molar mass [13,14,15]. The relative amount of hydroxyl groups functionalized with cationic groups is usually reported as the degree of substitution (DS). In the paper industry, usually low DS starches ranging from 0.02 to 0.1 are employed. These serve as an adsorbate on the negatively charged cellulose fibers, improve the fixation of other additives [16,17], increase strength, improve abrasion resistance, and contribute to maintain dimensional stability [18]. The efficiency of cationic starches as strength enhancing agents is correlated with the amount adsorbed onto cellulosic fibers [19,20]. The interaction of cationic starch with cellulosic materials and thin films has been extensively studied [21,22,23,24]. The amount of starch that adsorbs to the fibers is affected by various factors, related to the source and treatment of the pulp. Recovered pulp shows differences in starch retention compared to Kraft pulp [23,25]. The fine content of the pulp also has a strong influence on the starch retention. Fines can adsorb starch amounts of up to 250 mg/g, whereas 7.5 mg/g is adsorbed by cellulosic fibers [24]. The dependency on the surface area of the pulp can be determined by studying cellulose thin films. Cationic starch adsorption to cellulose thin films follows the general trends of polyelectrolyte adsorption [22] with quantities of up to 4 mg/m^2^ of industrial relevant cationic starches adsorbing to the surface of cellulose thin films which is a 2D-system [21]. Although such experiments give insights into cellulose-starch interactions, the results are not directly transferable to papermaking processes for several reasons. An important parameter in this context is the solubilization method, which impacts the interaction behavior with cellulose materials. In most studies in literature, solubilization was accomplished on a heating plate which gives solutions with rather different behavior than those obtained by industrially employing steam-jet cooking [26,27] in which a high-velocity steam jet is mixed with a starch-water-slurry, heating it up rapidly. Temperatures of more than 120 °C are commonly applied, often combined with elevated pressures (2–6 bars). Additionally, shear is applied which supports complete dissolution of the starch. Typically, starch-slurries are cooked at high concentrations (up to 15 wt.%), diluted to the final concentration and subsequently added to the cellulose-fibers. The exact process parameters differ depending on the produced paper product, dosage point, and intended function of the starch. Determination of the adsorbed starch content, also termed starch retention, is therefore an important aspect in the production of paper, as non-retained starch may lead to an increased effluent load and causes additional costs. The properties of cationic starch itself as well as the cooking conditions can have a significant impact on the adsorption behavior. The cooking temperature is another economically interesting parameter for industry. By decreasing the temperature, energy input and in that way costs as well as the environmental footprint can be reduced.

In the current study, the effect of cooking parameters on the adsorption efficiency of cationic starch on cellulosic fibers was investigated. A jet-cooking procedure comparable to the process used in the paper industry was used. Four starches used in the paper industry, differing in the cationization process (wet and dry), degree of substitution (DS), and source (potato and corn), were studied. Their adsorption behavior on pulp fibers was investigated. Experiments were performed at different cooking temperatures, focusing on determining the lowest temperature necessary to achieve maximum cationic starch retention. As the cooking procedure resembles the process used in the paper industry, the results will give an understanding of how the cooking conditions affect the adsorption behavior.

## 2. Materials and Methods

### 2.1. Materials

Wet-cationized potato starch P-W1 and P-W2 were obtained from Südstärke GmbH (Schrobenhausen, Germany); dry cationized potato (P-D) and corn (C-D) starches were obtained from Roquette Frères (Lestrem, France). The experiments were performed using dried, bleached, unrefined birch, and aspen wood Kraft pulp (fines content 16%, 15°SR (Schopper-Riegler), cationic demand 4 meq/g) provided by a local pulp mill in Austria.

Sodium chloride and potassium chloride were purchased from Herba Chemosan Apotheker-AG (Vienna, Austria) (purity: 99.97%) and Fluka Analytical (purity: >99%), respectively. Iodine–potassium iodide solution acc. to Lugol was obtained from Carl Roth GmbH + Co. KG (Karlsruhe, Germany).

The nitrogen content of the samples was determined by elemental analysis using a CNHS Elemental Analyzer, Vario MICRO Cube (Elementar Analysensysteme GmbH, Langenselbold, Germany). Starch samples were dried for 24 h at 100 °C before the measurements. From the resulting percentage nitrogen content (*x*(*N*)), the degree of substitution (*DS*) was calculated according to following formula:

(1) $$DS\;=\;\frac{x\left(N\right)\times M_{r}\left(anGlu\right)}{M_{r}\left(N\right)\times 100-M_{r}\left(GTAC\right)\times x\left(N\right)}$$

where *M_r_*(*anGlu*), *M_r_*(*N*), and *M_r_*(*GTAC*) are the relative molecular weights of anhydroglucose, nitrogen, and glycidyltrimethylammonium chloride, respectively. The results for the different starches are depicted in Table 1.

### 2.2. Methods

#### 2.2.1. Cooking of Cationic Starches with a Steam Jet Cooker

**Figure 1:**
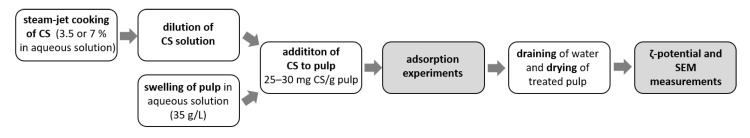
Flowchart of the experimental methods applied to cationic-starch (CS).

The cationic starch powder was placed into a 25 L plastic canister, and 5 L of deionized water containing KCl (40 mg/L) and NaCl (120 mg/L) was added. The mixture was shaken vigorously to ensure a homogenous distribution of the starch. After transferring the starch slurry into the storage vessel of the steam jet cooker (capacity 50 L), it was stirred at 50 rpm with a EUROSTAR 20 stirrer (IKA^®^-Werke GmbH & CO. KG, Staufen, Germany) to prevent sedimentation. It was attached to a Netzsch Mohno pump (type NM021BY02S12B, NETZSCH Pumpen & Systeme GmbH, Waldkraiburg, Germany) with flow rates reaching up to 50 L/min. The pressure of the slurry inlet was controlled by a Jordan GP valve (Jordan Valve, Cincinnati, OH, USA). The steam was generated by a steam generator (PONY GE20/04P, PONY S.p.A., Inzago, Italy) which generates pressures ranging from 0.1 to 6 bars. In order to keep the aperture running smoothly, the pressure was always kept below 3 bars. In a hydro THERMAL M101 hydroheater reaction chamber (Hydro-Thermal Corporation, Waukesha, WI, USA), the steam was mixed with the starch slurry inlet stream. Pressure and temperature could be changed via a control panel. The flow rate was set to 6 L/min at a pump frequency of 9.7 Hz. The temperature used to cook the starch slurry was varied between 115 and 130 °C, dependent on the experiment. The first few hundred milliliters were discarded, and then the cooked starch solutions were transferred to insulated bottles. A flowchart in Figure 1 displays the workflow of the experimental methods.

#### 2.2.2. Adsorption Experiments

Pulp panels (35 g) were cut in small pieces (<2 cm × 2 cm), equilibrated in 1 L of deionized water containing KCl (40 mg/L) and NaCl (120 mg/L) (conductivity of approximately 30 mS/m), and stirred for 24 h with a KPG stirrer at room temperature to guarantee complete swelling of the unrefined pulp fibers. Then, 250 mL of water was removed from the beaker, and a sample was taken for background determination. The cooked starch solution was diluted with the electrolyte solution to give a final concentration of 3.5 to 4.2 g/L and stirred with a magnetic stirrer at 500 rpm for 60 min at room temperature. A sample of the diluted starch solution was taken for analysis; 250 mL of the diluted starch solution was added to the pulp and mixed throughout the experiment at 500 rpm with a KPG stirrer. Samples were taken after 1, 5, 10, and 15 min. The final ratio of starch to pulp was 25 to 30 mg/g in all the adsorption experiments, which is the upper limit of cationic starch used in industrial applications. The experiments were performed in triplicate. After the adsorption experiments, the starch solution was drained with a sieve, and the pulp was squeezed by hand to remove any excess solution. A petri dish was filled with the pulp samples which were allowed to dry at room temperature. These samples were subject to scanning electron microscopy and ζ-potential measurements. For comparison, pure pulp was treated the same way.

#### 2.2.3. Determination of the Amount of Cationic Starch Adsorbed to Cellulosic Pulp

The amount of starch which was adsorbed to the pulp was determined by measuring the remaining starch concentration in the solution. Samples were taken 1, 5, 10, and 15 min after the starch solution was added to the pulp. The samples were diluted to obtain starch concentrations between 10 to 100 mg/L; 2.7 mL of starch solution was put into a polystyrene cuvette, and 0.3 mL of Lugol solution was added. For the measurement, a UV/VIS Spectrometer “Lambda 25” (PerkinElmer, Waltham, MA, USA) was used. The measurement was made from 450 to 800 nm with a scan speed of 480 nm/min in absorption mode using a 1 nm slit. The data were collected with the software “Lambda 25”. Starch concentrations were calculated by Lambert–Beer’s Law:

(2) $$c\;=\;\;\frac{A_{660nm}}{\varepsilon_{660nm}*l}$$

where *c* is the concentration of starch in mg/mL, *l* is the optical path length, which was 1 cm in all measurements, *A*_660*nm*_ is the absorbance at 660 nm, and $\varepsilon_{660\mathrm{nm}}$ is the respective extinction coefficient determined by measurement of calibration lines at 660 nm (Table 2).

#### 2.2.4. Amylose Content

The amylose content was estimated using the method described by Hovenkamp-Hermelink [28]. Starch solutions with a concentration of 2 mg/mL were prepared. Afterwards, 2.7 mL of these solutions were stained with 0.3 mL of Lugol solution. The absorbencies of the solutions were measured at 618 and 550 nm (*A_618nm_* and *A_550nm_*) using a UV/VIS Spectrometer “Lambda 25” (PerkinElmer, Waltham, MA, USA). Their ratio, (*R*) *A_618nm_*/*A_550nm_*_,_ was used to calculate the fraction of amylose (*P*) using following formula:

(3) $$P\;=\;\;\frac{3.5-5.1\times \;R}{10.4\times R-19.9}$$

#### 2.2.5. ζ-Potential of Pulp Fibers Treated with Cationic Starches

The ζ-potential measurements of pulp fibers were performed by an electrokinetic analyzer SurPASS^TM^ 3 (Anton Paar GmbH, Graz, Austria). Four measurements were done and averaged. The pulp was treated with potato starches and corn starch cooked by steam jet cooking at 115 and 125 °C, respectively. Approximately 0.5 g of the sample (prepared according to Section 2.2.2) and approximately 20 mL of aqueous KCl solution (1 mM) were mixed and allowed to equilibrate for 24 h. The aqueous solution was separated from the pulp fibers with a sieve. The fibers were filled in a cylindrical cell, and the streaming potential in 1 mM KCl solution was recorded. Measurements were done at pH values between app. 6.5 and 7.5, adjusted by the addition of aqueous NaOH solution (50 mM).

#### 2.2.6. ζ-Potential and Particle Size of Starches

For the characterization of starch samples, a laboratory method was used to prepare the starch solutions. The corresponding amount of starch and 200 mL of deionized water were placed into a glass reactor. The reactor was equipped with a glass tube immersed into the liquid and connected to a Kärcher Steam Cleaner SC1 easyfix (Alfred Kärcher GmbH, Vienna, Austria) which produced steam to dissolve the starch. The steam cleaner was filled with 100 mL deionized water, and the steam was injected into the starch slurry until its tank was empty (approx. 3 min.). Afterward, the starch solution was homogenized using a hand blender (Severin, 170 W, 20,000 rpm, SEVERIN Elektrogeräte GmbH, Sundern, Germany) for one minute. The starch solutions were diluted with deionized water to obtain final concentrations of 1 mg/mL for particle size and ζ-potential measurements as well as for the determination of the conductivity of the starch solutions. All the following experiments were performed in triplicate. The hydrodynamic radius, conductivity, and ζ-potential were measured at 25 °C using a Litesizer 500 (Anton Paar GmbH, Graz, Austria). Starch samples containing 1 mg/mL starch in deionized water were filled into cuvettes and allowed to equilibrate at 25 °C for at least five minutes before the measurement. Kalliope^TM^ software was used for measurement and data evaluation. The cumulant model ISO 22,412 was used for the calculation of the mean hydrodynamic diameter. The analysis of the ζ-potential was performed by applying the Smoluchowski approximation with a Debye factor of 1.50.

#### 2.2.7. Scanning Electron Microscopy of Cellulosic Pulp Containing Cationic Starch

Scanning electron microscopy (SEM) images of gold-sputtered samples were obtained with a TESCAN VEGA3 (TESCAN, Brno, Czechia).

## 3. Results

### 3.1. Preparation of Solutions and Setup of the Adsorption Experiment

Different types of steam-jet cooked cationic starches were adsorbed on cellulosic pulps. The starches (two potato and two corn starches) featured a DS between 0.037 and 0.055 and were either dry or wet cationized. These starches were subjected to dissolution via steam-jet cooking in deionized water with defined ionic strength that matches typical conditions present in process waters. The temperature (115, 120, 125, 130 °C), as well as the concentration of the starches (3.5 and 7 wt.%) were varied and their influence on the adsorption behavior investigated. Before addition to the pulp fiber suspension, the starch solutions were diluted. Retention was monitored and quantified by visible light absorption at 660 nm of the starch–iodine complex. Adsorption times were varied between 1 and 15 min as these are typical times for starch adsorption in an industrial context. The experiments were performed at relatively high starch-to-pulp ratios.

### 3.2. Retention of Different Cationic Starches on the Fibers as a Function of Adsorption Time

**Figure 2:**
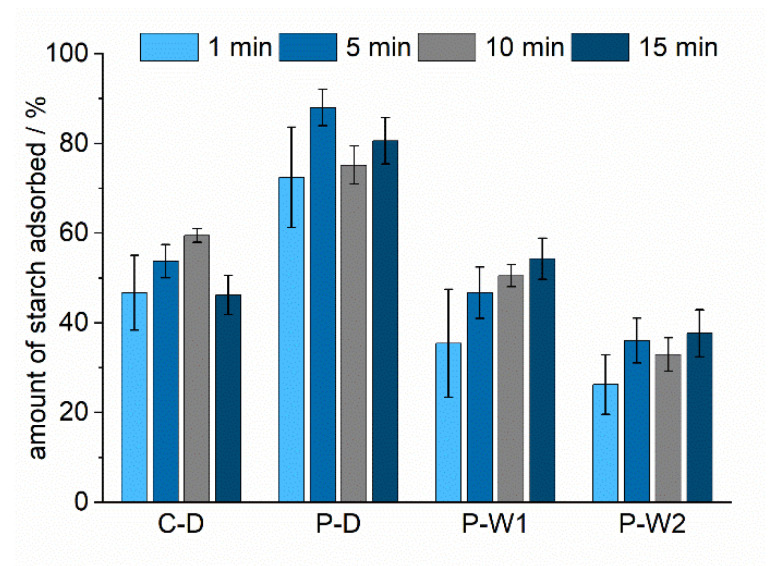
Adsorption efficiencies of different cationic starches (C-D: dry-cationized corn starch, P-D: dry-cationized potato starch, P-W1: wet-cationized potato starch, P-W2 wet-cationized potato starch with higher DS) cooked by steam-jet cooking (125 °C, 3.5 wt.%).

The starches were cooked at 125 °C at a concentration of 3.5 wt.%. Subsequently, the influence of adsorption time on the adsorption efficiency, i.e., how much of the starch was retained on the fibers, was explored. A starch-to-pulp ratio of 25 to 30 mg/g was chosen in the experiments, and the adsorption time was varied between 1 and 15 min. Most of the starch was already adsorbed within the first minute of the experiment. Dry-cationized corn and potato starches showed a significant reduction of adsorbed material between 10 and 15 min and between 5 and 10 min, respectively. This phenomenon was related to the replacement of adsorbed starch clusters by single starch molecules [29]. The results showed that longer adsorption times did not necessarily lead to maximum retention on the fibers (Figure 2). P-D showed a higher retention on the pulp than the other starches at all adsorption times, with a maximum after 5 min (88%). After 15 min, retention slightly decreased again (81%); 60% of C-D was retained after 10 min with a decrease to 46% after 15 min adsorption time. The other starches, PW-1 and PW-2, cooked at 125 °C, exhibited a maximum retention at 15 min of 54% and 38%, respectively. The results of adsorption experiments after 15 min were used for comparison to ensure uniform distribution of starch and pulp. Although the adsorption efficiency may increase with longer adsorption times, these are not relevant for industrial applications.

The starch retentions translate to maximum adsorption of 10 to 24 mg starch/g of pulp (Table 3). Assuming a surface area of 1.5 m^2^/g according to [30] for unrefined pulp, the surface concentration (Γ) of starch on the fibers was determined, yielding values between 7–16 mg/m^2^.

### 3.3. Effect of Cooking Temperature on Adsorption Behavior

**Figure 3:**
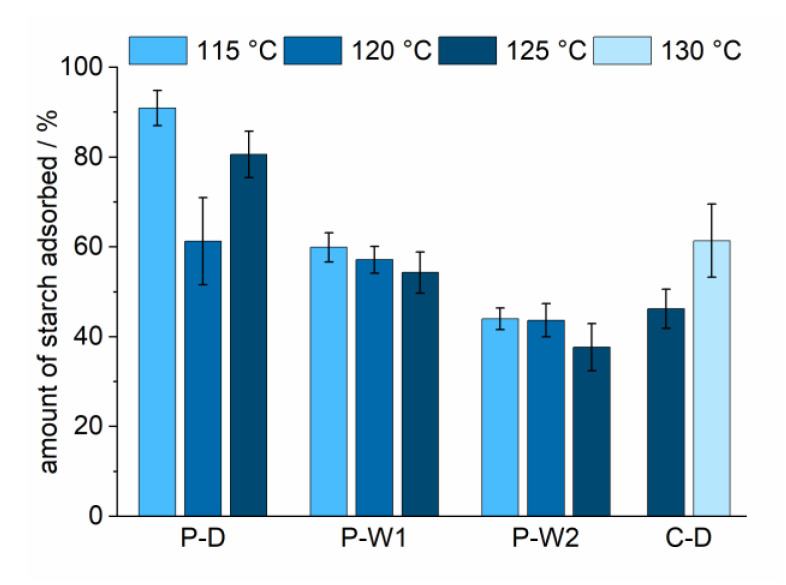
Adsorption efficiency of the starches as a function of cooking temperature (C-D: dry-cationized corn starch, P-D: dry-cationized potato starch, P-W1: wet-cationized potato starch, P-W2 wet-cationized potato starch with higher DS).

In industry, the cooking temperature and concentration are directly correlated with the energy consumption of the starch cooking process. A decrease in cooking temperature is therefore an important factor in reducing energy costs. Steam-jet cooking temperatures of 120 and 115 °C were applied for the three potato starches. Adsorption time was set to 15 min in this set of experiments. The potato starches showed a clear increase in adsorption efficiency when the cooking temperature was reduced from 125 to 115 °C (Figure 3). The effect was most pronounced for the dry-cationized sample P-D, showing significantly higher adsorption values at a lower cooking temperature. The cationic corn starch (C-D) could not be properly processed at temperatures lower than 125 °C using steam-jet-cooking. To see the effect of temperature on retention, the temperature was therefore increased to 130 °C. For this type of starch, an increase in temperature induced higher adsorption.

### 3.4. Effect of Starch Concentration during Cooking on Adsorption Behavior

**Figure 4:**
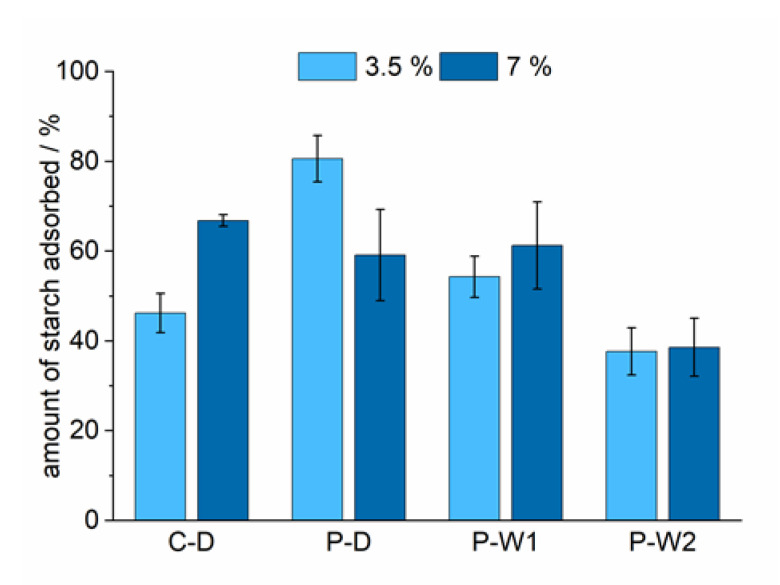
Adsorption efficiency depending on the type of cationic starch (C-D: dry-cationized corn starch, P-D: dry-cationized potato starch, P-W1: wet-cationized potato starch, P-W2 wet-cationized potato starch with higher DS) and concentration cooked by the steam-jet method at 125 °C. The final ratio of starch to pulp was 25–30 mg/g pulp in all the adsorption experiments.

The energy input for the dissolution of starch in the paper industry can further be reduced by jet-cooking increased starch concentrations. The experimental conditions of the adsorption experiments were kept the same, only the concentration of starch during the cooking procedure was doubled. The increased concentration caused a significantly increased adsorption efficiency for dry-cationized corn starch (C-D) (Figure 4). However, the adsorption of dry-cationized potato starch (P-D) was reduced when it was cooked at higher concentrations. Wet-cationized potato starches P-W1 and P-W2 did not show major differences in adsorption when the cooked concentration was increased.

### 3.5. Amylose/Amylopectin Ratio

Different absorption maxima of the iodine complexes of amylose and amylopectin enabled the observation of changes in composition during the adsorption experiments. The absorption maximum of the starch remaining in solution shifted during the adsorption. This indicated that amylopectin and amylose were not adsorbed to the same extent. The shift of the absorption maximum to lower wavelengths indicated that the ratio in the solution changed to higher amylopectin contents. In turn, there was an increased amount of amylose adsorbed onto the pulp fibers. The amylose content in the drain water was estimated according to Hovenkamp-Hermelink [28] (Table 4). P-D had the highest amylose content, the compositions of P-W1 and P-D were comparable, and P-W2 showed the lowest amylose fraction. Comparing these results to the values observed after the adsorption experiments showed a reduction of the amylose content in the drain water to a different extent for all the samples. Although the amylose/amylopectin content varied depending on the sample, the amylose content in the drain water was similar. These results reflect the preferential adsorption of amylose [29,31] and also explain the higher adsorption efficiency for starches with higher amylose contents.

### 3.6. ζ-Potential of Cationic Starches and Pulp Fibers Treated with Cationic Starches

The electrostatic interaction of cationic starches with cellulosic fibers is related to their charges. As a measure of the effective charge on the surface, the ζ-potential of starches in pure water was determined (Table 5). The ζ-potential is the potential occurring at the shear plane of cationic starches and is very sensitive to electrolytes present in the solution. In the dry-cationization process, unreacted cationization agents and byproducts remain in the sample. Comparing potato starches functionalized by wet-cationization showed an expected increase of ζ-potential caused by higher density of charges for higher DS. On the contrary, ζ -potentials observed for dry-cationized samples were lower. These charged species increased the ionic strength in the solution and thus induced a reduction of the ζ-potential. The presence of charged species in dry-cationized starches was validated by the conductivity of the starch solutions. Although the DS values are similar, interaction with cellulosic materials could be reduced due to the reduced apparent charge of starches caused by the presence of those electrolytes. Additionally, charges on the polymer chains influenced their conformation in solution. Repulsive interactions of charged residues induced expansion of the polymer chain and resulted in less compact conformations (Table 5). Comparing P-W1 and P-W2, an increase in particle size was observed for the starch with the higher DS, resulting in higher charge densities on the polymer chain.

**Figure 5:**
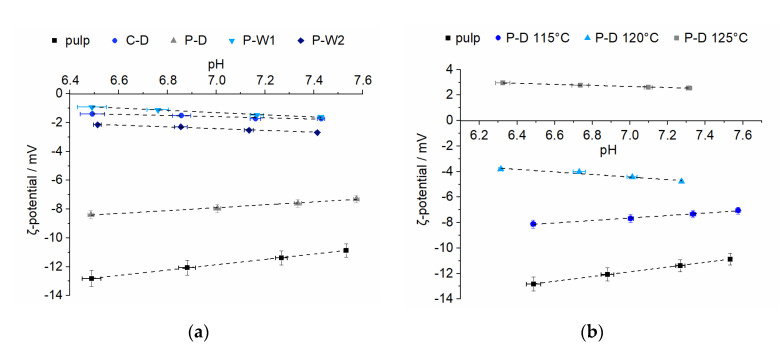
ζ-potential of pure pulp fibers (pulp) and fibers treated with steam-jet cooked cationic starches: (**a**) potato (P-D, P-W1, P-W2) and corn (C-D) starches cooked at 115 and 125 °C, respectively; (**b**) dry cationized potato starch (P-D) cooked at 115, 120, and 125 °C.

In order to evaluate the effect of cationic starch on the charge properties of pulp, the ζ-potentials of untreated pulp as well as those treated with different starches were determined. All samples showed a reduction of the negative charge on the fibers (Figure 5a). The charge was almost eliminated when the fibers were treated with wet-cationized potato and dry-cationized corn starches. Although P-W2 had a higher DS, comparable ζ-potentials were observed for fibers treated with P-W1 and P-W2 due to the lower amount of P-W2 adsorbed to the fiber. Interestingly, the fiber containing dry-cationized potato starch, which showed the highest adsorption efficiency, had the lowest impact on the ζ-potential. In the case of the most effective adsorbent P-D, the effect of the steam-jet cooking temperature was also investigated (Figure 5b). The ζ-potential of the fibers increased with increasing steam-jet temperature, resulting in a positive charge for fibers treated with starch P-D that was cooked at 125 °C. Although less starch adsorbed when it was cooked at this temperature, the starch was able to more effectively screen the pulp’s negative charge compared to starch cooked at 115 °C. This indicated that the starch was spread more evenly on the surface of the fiber. The dissolution state and subsequently the distribution of the starch on the fiber affected the resulting ζ-potential. Comparable ζ-potentials between −1 mV and −5 mV were observed for pulp containing P-W1 and P-W2 cooked at 115 °C, P-D cooked at 120 °C, and C-D cooked at 130 °C.

### 3.7. Scanning Electron Microscopy (SEM) of Cellulosic Pulp with Adsorbed Cationic Starches

**Figure 6:**
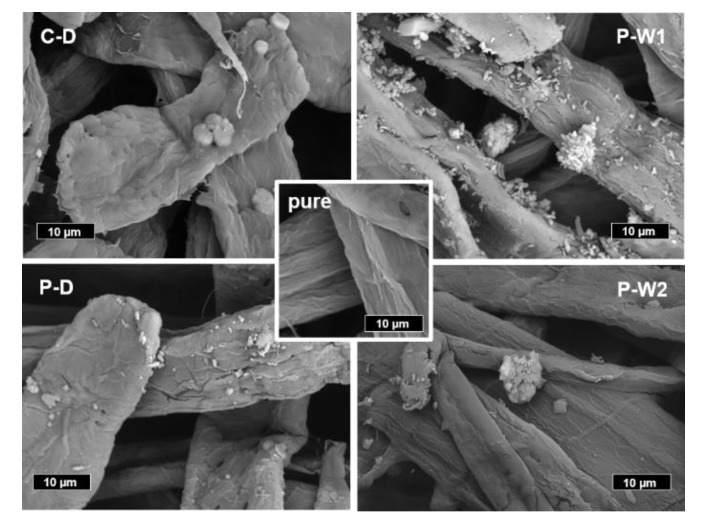
Scanning electron microscopy pictures of pulp fibers with adsorbed cationic starches (C-D: dry-cationized corn starch, P-D: dry-cationized potato starch, P-W1: wet-cationized potato starch, P-W2 wet-cationized potato starch with higher DS, pure: pristine cellulosic pulp).

Pulp fibers with adsorbed starches were dried and monitored by SEM. For all samples, starch adsorbed to the pulp was observed (Figure 6). The appearance of the samples displayed different distributions of starch on the fibers. Beside smaller particles, large starch aggregates were observed on all fibers. Mainly larger aggregates were detected in the case of corn starch whereas a large number of smaller particles was discovered on the fibers treated with P-W1 and P-D.

## 4. Discussion

Four different, industrially relevant cationic starches were studied. The starches differed in source (corn and potato) and cationization process (wet and dry) and had a slightly different degrees of substitution. Depending on the type of starch, different retention efficiencies were observed. Determination of the amylose content (Table 4) revealed that P-D has higher portions of amylose than the other starches. Amylose has a higher tendency to adsorb to the pulp fiber compared to the highly branched amylopectin because it can diffuse into the pulp fiber pores [29]. The higher affinity of amylose to the pulp and the significant higher portion of amylose in P-D could be the reason for its high adsorption efficiency. The similar amylose content of C-D and P-W1 resulted in comparable adsorption efficiencies. Beside variations in amylose content, differences in effective charge could further affect adsorption efficiencies. Differences related to variations in DS could be deduced from comparing potato starches. The DS determined for potato starches was lowest for the dry-cationized one (P-D), intermediate for P-W1, and the highest for P-W2. Results from ζ-potential measurements support these findings (Table 5). Starches containing higher charge densities resulted in decreased adsorption efficiencies. The electrostatic repulsion of cationic groups along the polymer caused a more extended conformation of the starch molecules. This extended conformation and the higher amount of charges on each molecule resulted in a more efficient screening of the negative charge of the pulp. As a consequence, less starch was required to compensate for the anionic charge of the pulp fibers which was observed as a lower adsorption efficiency. The time dependence of cationic starch adsorption was studied to show the kinetics of the adsorption process (Figure 2) and revealed that most of the starch was already adsorbed after one minute. Beside variations in the affinities of different cationic starches, the study focused on the evaluation of the effect of starch cooking conditions on the adsorption behavior. The focus was put on the reduction of the energy consumption due to a reduction of the cooking temperatures and increased cooked starch concentrations. All potato starches tested showed increased adsorption efficiencies when the cooking temperature was reduced from 125 to 115 °C (Figure 3). Increased adsorptions for corn starch cooked at 130 °C compared to 125 °C were related to an incomplete dissolution of corn starch at 125 °C. An additional reduction in energy consumption may also be achieved by increasing the starch concentration for the cooking process. The increase in the concentration of cooked starch is a feasible approach to reduce the energy needed for the dissolution of dry-cationized corn starch (C-D) and wet-cationized potato starches (P-W1 and P-W2) (Figure 4). This even showed a positive effect on the adsorption efficiency of C-D and PW-1. A higher concentration of starch could influence the disruption of starch granules and could support the dissolution process. The starch components amylose and amylopectin show different adsorption behaviors on cellulosic substrates [29,31]. Knowledge of the relative amylose content is therefore crucial in understanding the adsorption behavior and comparison of different cationic starches. The ratio of amylose to amylopectin can be studied by various methods [32]. Many of them utilize colorimetry of starch–iodine complexes. The complexes with amylose and amylopectin show absorption maxima in the visible regime at 605 and 530 nm, respectively. In the current research, the method developed by Hovenkamp-Hermelink [28] was chosen. A drawback of iodine-based binding assays is that they tend to overestimate the amylose content [33]. Additionally, they are sensitive to various factors [34,35]. Although more accurate measurements do exist [36,37,38], these fast and easy measurements were chosen with the aim to point out relative differences in composition rather than absolute values. Photometric studies of the starch–iodine complex before and after the adsorption experiments revealed that amylose and amylopectin were not adsorbed to the pulp to the same extent (Table 4). The smaller amylose molecule adsorbed preferentially on the fibers for all tested starches. During adsorption, the amylose fraction remaining in the drain water was reduced to a higher extent for starches with higher amylose contents. This resulted in similar amylose/amylopectin ratios in the drain water of the adsorption experiments and also reflected the higher adsorption efficiency of starches with higher portions of amylose. Cationic starches could almost eliminate the negative charge of the cellulosic pulp fibers (Figure 5). The ζ-potential of starch-containing fibers showed interesting results regarding the effect of the cooking temperature in the case of dry-cationized potato starch (P-D). With increasing cooking temperature, the ζ-potential increased. This indicated that P-D cooked at higher temperature could distribute more uniformly on the surface of the pulp fibers.

## 5. Conclusions

In the current study, the adsorption behavior of four different industrially relevant cationic starches to cellulosic pulp was studied. The starches were pretreated by a steam-jet cooking method which is comparable to the cooking process used in the paper industry. The presented results have improved relevance to industrial applications contrary to experiments performed with laboratory cooking methods. The adsorption efficiency is related to the amylose content of the starches as well as their degree of substitution. A higher amylose content results in increased adsorption whereas the amount of adsorbed starch decreases with increasing degree of substitution. Besides these differences, the adsorption of the cationic starches is also affected by the cooking conditions. Current results suggest that the cooking temperature of cationic potato starch can be reduced to 115 °C without a reduction of adsorption efficiency. Additionally, an increased concentration only negatively affects the adsorption of dry-cationized potato starch, whereas the adsorption of dry-cationized corn starch increases. A reduction of cooking temperature as well as an increase in starch concentration during the cooking procedure significantly reduced the energy consumption of this process.

## Figures and Tables

**Table 1:** Nitrogen content and degree of substitution (DS) (experimental results and values provided by the suppliers) of four different cationic starches used in the paper industry.

Origin	Cationization	Supplier	DS/N-Content Supplier	N-Content Experimental	DS Experimental	Sample ID
Corn	dry	Roquette	n.a./0.35–0.4%	0.42%	0.051	C-D
Potato	dry	Roquette	n.a./0.35–0.4%	0.31%	0.037	P-D
Potato	wet	Südstärke	0.035/n.a.	0.36%	0.043	P-W1
Potato	wet	Südstärke	0.055/n.a.	0.45%	0.055	P-W2

**Table 2:** Extinction coefficients at 660 nm of iodine starch complexes of four cationic starches.

Starch	$\varepsilon_{660\mathrm{nm}}$ /mL mg^−1^ cm^−1^
C-D	8.295
P-D	5.948
P-W1	12.072
P-W2	8.574

**Table 3:** Amount of different steam-jet cooked (125 °C, 3.5%) cationic starches adsorbed to cellulosic pulp after 15 min.

Starch	Starch/Pulp mg/g	Γ_starch_ mg/m^2^
C-D	13.6 ± 1.3	9.1 ± 0.9
P-D	24.1 ± 1.6	16.1 ± 1.0
P-W1	13.4 ± 1.1	8.9 ± 0.7
P-W2	10.4 ± 1.5	7.0 ± 1.0

**Table 4:** Amylose content of cationic starch solutions before and after adsorption to pulp fibers.

Starch	Amylose Content before Adsorption to Pulp/%	Amylose Content after 15 min Adsorption to Pulp/%
C-D	17.7 ± 0.6	12.2 ± 1.8
P-D	21.2 ± 0.1	11.8 ± 1.2
P-W1	18.3 ± 0.6	12.5 ± 4.3
P-W2	12.6 ± 0.5	10.8 ± 0.1

**Table 5:** Hydrodynamic diameters, ζ-potentials, and conductivities of aqueous starch solutions containing 1 mg/mL cationic starch.

Starch	ζ-Potential/mV	Conductivity/mS/cm	Hydrodynamic Diameter/nm
C-D	+15.2 ± 0.1	0.068	250.5 ± 5.6
P-D	+15.2 ± 0.2	0.062	274.2 ± 8.6
P-W1	+21.8 ± 0.9	0.043	381.5 ± 2.0
P-W2	+30.1 ± 0.5	0.031	428.7 ± 13.5
